# Separated parabiont reveals the fate and lifespan of peripheral-derived immune cells in normal and ischaemia-induced injured kidneys

**DOI:** 10.1098/rsob.200340

**Published:** 2021-06-09

**Authors:** Xuan Deng, Cheng Zhou, Ruichun Liao, Yi Guo, Yuxi Wang, Guoli Li, Jianliang Wu, Huzi Xu, Zhizhi Hu, Guangchang Pei, Wenhui Liao, Ying Yao, Qian Yang, Rui Zeng, Gang Xu

**Affiliations:** ^1^ Division of Nephrology, Huazhong University of Science and Technology, 1095 Jiefang Ave, Wuhan, Hubei 430030, People's Republic of China; ^2^ Geriatrics, Tongji Hospital, Tongji Medical College, Huazhong University of Science and Technology, 1095 Jiefang Ave, Wuhan, Hubei 430030, People's Republic of China

**Keywords:** immune cells, acute kidney injury, chronic kidney disease, parabiosis, lifespan

## Abstract

Immune cell infiltration plays a key role in acute kidney injury (AKI) to chronic kidney disease (CKD) progression. T lymphocytes, neutrophils, monocytes/macrophages and other immune cells regulate inflammation, tissue remodelling and repair. To determine the kinetics of accumulation of various immune cell populations, we established an animal model combining parabiosis and separation surgery to explore the fate and lifespan of peripheral leucocytes that migrate to the kidney. We found that peripheral T lymphocytes could survive for a long time (more than 14 days), whereas peripheral neutrophils survived for a short time in both healthy and ischaemia-induced damaged kidneys. Nearly half of the peripheral-derived macrophages disappeared after 14 days in normal kidneys, while their existing time in the inflammatory kidneys was prolonged. A fraction of F4/80^high^ macrophages were renewed from the circulating monocyte pool. In addition, we found that after renal ischaemia reperfusion, neutrophils increased significantly in the early phase, and T lymphocytes mainly accumulated in the late stage, whereas macrophages infiltrated throughout AKI-CKD progression and were sustained longer in injured as opposed to normal kidneys. In conclusion, peripheral-derived macrophages, T lymphocytes and neutrophils exhibit different lifespans in the kidney, which may play different roles during AKI-CKD progression.

## Graphical abstract

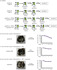

## 1. Introduction

Acute kidney injury (AKI) is a common disease defined as a rapid loss of renal excretory function, leading to the accumulation of nitrogenous waste products [1,2]. Development or progression of chronic kidney disease (CKD) following one or more episodes of AKI has resulted in striking public health and socioeconomic consequences [3,4]. Although some patients with mild AKI present recovered renal function after proper treatment, AKI in most patients causes long-term renal damage, inducing chronic inflammation and renal fibrosis, which eventually lead to CKD, end-stage renal disease and death [5,6]. However, no effective drug has emerged that can prevent AKI or reverse the AKI-CKD progression [7].

The aetiology of AKI would be septic or aseptic (ischaemic or nephrotoxic) [8], and its common pathological features are cell death, inflammation, and fibrosis [7]. After the occurrence of AKI, necrotic tubular cells release intracellular molecules to activate pattern recognition receptors on the resident and the recruited immune cells [9–11], which then secrete pro-inflammatory cytokines and chemotactic factors [12]. These inflammatory responses further stimulate immune cells to enter the injured kidney, causing more cell death [13,14]. These immune cells including T lymphocytes, neutrophils and monocytes/macrophages regulate the inflammatory response as well as tissue remodelling and repair in the injured kidney [15,16].

It is generally believed that T lymphocytes, neutrophils and monocytes/macrophages are derived from haematopoietic progenitors [17–19]. Before encountering cognate antigens, T lymphocytes may circulate between different tissues for a long time, ranging from months to years [20], whereas neutrophils are generally considered to have a short life cycle [21]. Macrophages, due to their high heterogeneity and plasticity, exhibit different lengths of life cycle according to different sub-types and settled tissues [22]. However, the survival of immune cells after the renal injury is still unclear.

The survival status of various immune cells changes dynamically and presents different roles in different phases of kidney injury. Few studies have reported the survival and prognosis of peripheral immune cells after migrating to the kidney. Therefore, we established a model of parabiosis combining with ischaemia reperfusion injury (IRI) and subsequent separation, and tracked the peripheral immune cells that migrated to the injured kidney in order to figure out the destiny of intra-renal haematopoietic immune cells at different stages after renal injury and the potential therapeutic target.

## 2. Material and methods

### 2.1. Animals

C57BL/6-Tg (CAG-EGFP) mice were provided by Jackson Laboratory, USA (JAX006567). Healthy male C57BL/6 mice were purchased from Hua Fukang Experimental Animal Center (Beijing, China). All mice were housed in a specific pathogen-free (SPF) environment with a 12 h light-dark cycle. The room temperature was maintained at (22 ± 2)°C. Food and water were freely available. All the mice used in the experiment were aged 9–10 weeks and weighed 24–26 g. We randomly paired wild-type (WT) male mice with GFP mice. The paired two mice were about the same size and placed in the same cage for at least one week before subsequent operations. The mice in pairs were randomly divided into the following groups. (i) The paired mice were symbiotically attached for four weeks, after which the WT mice did not undergo IRI (parabiosis + IRI d0, *n* = 4). (ii) The paired mice were symbiotically attached for four weeks, after which the WT mice were subjected to IRI (parabiosis + IRI d3, *n* = 6). (iii) After four weeks of parabiosis, pairs were surgically separated for 14 days (parabiosis + IRI d0 + separation d14, *n* = 6). (iv) After four weeks of parabiosis, the WT of the paired mice underwent IRI, and then the parabionts were surgically separated for 14 days (parabiosis + IRI d3 + separation d14, *n* = 7). The left kidneys from WT mice in paired mice were harvested for subsequent experiments.

### 2.2. Parabiosis

The surgical operation of parabiosis was performed based on the method conducted by Paniz Kamran [23]. Paired mice were anaesthetized with 1% sodium pentobarbital solution (8 µl g^−1^, Sigma, USA) by intraperitoneal injection. After skin preparation and disinfection on the right side of WT mice and the left side of GFP mice, a longitudinal skin incision was made in each mouse from 0.5 cm above the elbow to 0.5 cm below the knee. Keeping the skin up, we gently separated the skin from the subcutaneous fascia with tweezers. The olecranon and knee joints of the two mice were tightly fixed together with non-absorbable sutures. The ventral skin of the incisions of two mice was connected using the interrupted exstrophy mattress suture method, then the skin on the dorsal side of the incision was sutured. Each mouse was injected subcutaneously with 0.5 ml of warm (37°C) 0.9% NaCl to prevent dehydration, and antibiotics were used at the surgical site to prevent infection.

### 2.3. IRI surgery

Parabionts were anaesthetized. Throughout the procedure, the body temperature was maintained between 36.8°C and 37.2°C using a sensitive temperature control machine (FHC, USA). The left renal artery of WT mice was occluded with a microvascular clip (Roboz Surgical Instrument Co, Germany) for 30 min, the left kidney turned black after clamping. After 30 min, the blood vessel clamp was removed, and the left kidney returned to red within about 10 s. Mice were allowed to wake up on a thermostatic blanket.

### 2.4. Seperation

The parabiotic pair anaesthetized with sodium pentobarbital solution was surgically separated using a midline incision. The non-absorbable surgical sutures joining the olecranon and knee joint of the two mice were removed. Separants were closed by suturing with absorbable 4-0 nylon sutures. Each mouse was given 0.5 ml of warm saline and appropriate amount of antibiotics.

### 2.5. Renal histopathology and immunofluorescence

Kidneys were fixed in 4% paraformaldehyde for 24 h, embedded in paraffin and cut into thin slices (4 µm). Periodic acid-Schiff (PAS) staining was carried out to assess renal pathological damage, and Masson staining was used to estimate the extent of tubular-interstitial fibrosis. The kidney tubular injury score was based on brush border loss, tubular dilation, cast formation, tubular cell death and detachment [24]. The kidney injury scores were evaluated blindly by two experienced kidney pathologists. For immunohistochemistry staining (IF) analysis, antigens were recovered by citrate buffer after deparaffinization and rehydration of samples. The renal sections were blocked with goat serum for 30 min and further incubated with antibodies at 4°C overnight: Kim-1 (1:1000, R&D system, USA), α-SMA (1:100, Abcam, UK), GFP (1:200, Abcam, UK), Ki67 (1:200, Abcam, UK), CD3 (1:50, Abcam, UK), F4/80 (1:200, Abcam, UK), Ly6G (1:100, Abcam, UK) and then visualized by fluorescent labelled secondary antibodies. The nuclei were stained with DAPI for 10 min. Data were analysed using the Image-Pro Plus 6.0 software (Media Cybernetics, Rockville, MD, USA) in more than eight random fields.

### 2.6. Flow cytometry

We prepared and stained single-cell suspension of kidneys as described in the previous report [25]. Antibodies were added to each tube of 100 µl single-cell suspension of kidneys and peripheral blood and incubated at room temperature for 30 min in the dark. Red blood cells were lysed by using lysis buffer for 5 min. The following antibodies were purchased from BioLegend: APC/Cy7–conjugated anti-mouse CD45 Ab; BV605-conjugated anti-mouse/human CD11b Ab; APC-conjugated anti-mouse F4/80 Ab; BV421-conjugated anti-mouse Ly6G Ab; BV421-conjugated anti-mouse CD3 Ab; PE-conjugated anti-mouse CD8 Ab; APC-conjugated anti-mouse CD4 Ab. The dead cells of kidneys were excluded by staining with Zombie dyes (Biolegend, catalogue no. 423101). Precision count beads (BioLegend, catalogue no. 424902) were used to obtain absolute counts of immune cells. The data were analysed by using FlowJo v. 10.0.7 software.

### 2.7. Quantitative real time-PCR

Total RNA was extracted from renal tissues using Trizol reagent (Takara, Japan) and cDNA was synthesized with the reverse transcription system-kit (Takara, Japan). Quantitative PCR was performed on Roche light 480II using SYBR master mix (Takara, Japan). Relative mRNA expression levels of IL-2, CSF-1 and IL-34 were calculated using the 2−ΔΔCt method and normalized to the expression levels of GAPDH. The following primer sequences for mice were used: IL-2, 5′-CCTTCAAATTTTACTTGCCCA-3′ (forward), 5′-TGAGTCAAATCCAGAACATGC-3′ (reverse); CSF-1, 5′-GTGTCAGAACACTGTAGCCAC-3′ (forward), 5′-TCAAAGGCAATCTGGCATGAAG-3′ (reverse); IL-34, 5′-TTGCTGTAAACAAAGCCCCAT-3′ (forward), 5′-CCGAGACAAAGGGTACACATTT-3′ (reverse); GAPDH, 5′-TTGATGGCAACAATCTCCAC-3′ (forward), 5′-CGTCCCGTAGACAAAATGGT-3′ (reverse).

### 2.8. Statistical analysis

Data were presented as mean ± s.d. ANOVA test was applied for comparison of more than 2 groups with GraphPad Prism 8.0.1. The statistical significance was expressed as follows: n.s., no significance; *p < 0.05; **p < 0.01; ***p < 0.001; ^#^*p* < 0.0001.

## 3. Results

### 3.1. Survival rates of paired mice in different animal models

**Figure 1.**
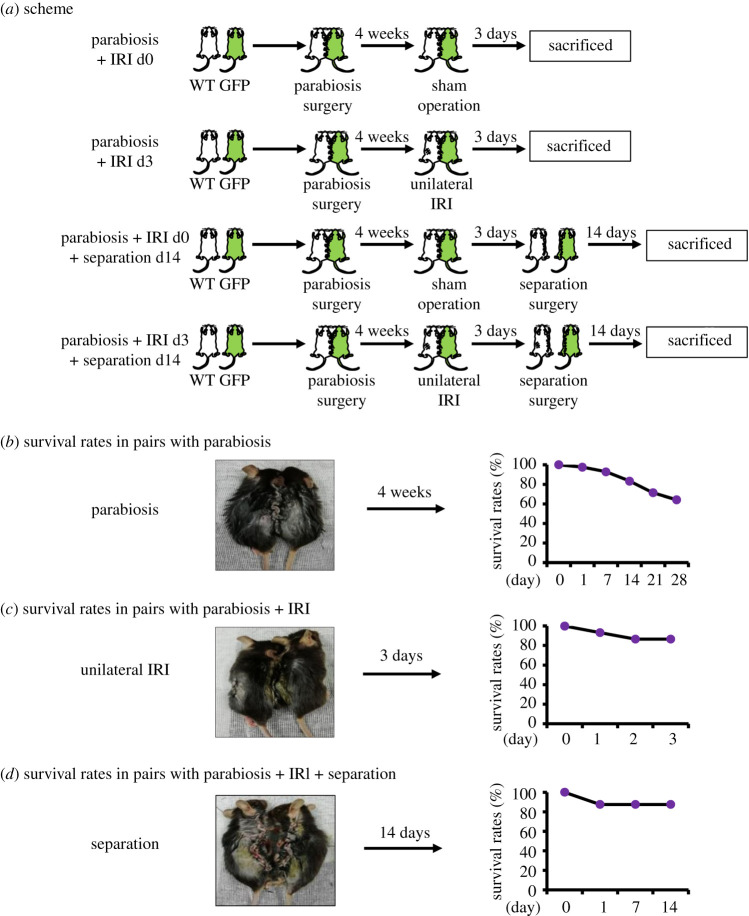
Survival rates of paired mice in different animal models. (*a*) Schematic of each model construction and experiment grouping. (*b*) The survival rate of paired mice in four weeks after parabiosis. (*c*) The survival rate of parabionts in 3 days after IRI. (*d*) The survival rate of paired mice in two weeks after separation surgery. Note: once one mouse dies, the pair is considered dead.

In order to explore the fate and lifespan of peripheral immune cells after they migrate into the kidney, we established a model including three kinds of operations: parabiosis, ischaemia reperfusion and separation. Paired mice of matching age and weight were divided into the following four groups: the parabiosis + IRI d0 group (uninjured parabionts); the parabiosis + IRI d3 group (injured parabionts); the parabiosis + IRI d0 + separation d14 group (uninjured separants) and the parabiosis + IRI d3 + separation d14 group (injured separants) (figure 1*a*). Conjoining operations were implemented in 42 pairs, with no intraoperative deaths. A total of 27 pairs survived on the 28th post-operative day, with a survival rate of 64.3%. Fifteen WT mice in the pairs underwent unilateral renal ischaemia for 30 min. Two pairs died at day 1 and day 2, respectively, after the operation, and the final survival rate was 86.7%. Eight pairs suffered three operations, only one of them died on the first day of post-separation, and the survival rate was 87.5% (figure 1*b*).

### 3.2. Successful establishments of parabiosis, separation and IRI models

**Figure 2.**
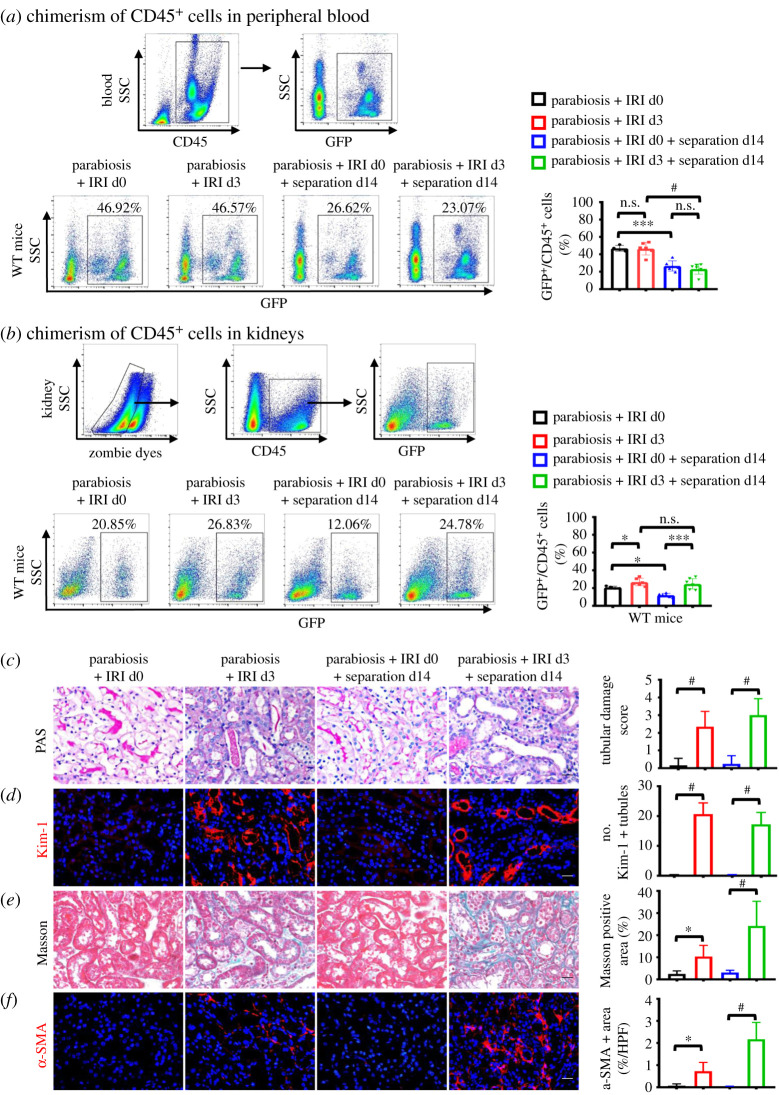
Successful establishments of parabiosis, separation, IRI models. (*a*,*b*) Percentage chimerism among CD45^+^ leucocytes by flow cytometry from the peripheral blood (*a*) and kidneys (*b*) of WT parabiotic partners. Percentage gated is shown for each region. (*c*,*d*) Representative images (left) and quantitative analyses (right) of PAS staining (*c*) and Masson Blue staining (*d*). (*e*,*f*) Representative images (left) and quantitative analyses (right) for immunofluorescence-labelled Kim-1 (red) (*e*) and a-SMA (red) (*f*). Scale bars = 20 µm. *n* = 4–7 per group. Values were means ± s.d. n.s., no significance; *p < 0.05; ***p < 0.001; ^#^*p* < 0.0001.

**Figure 3.**
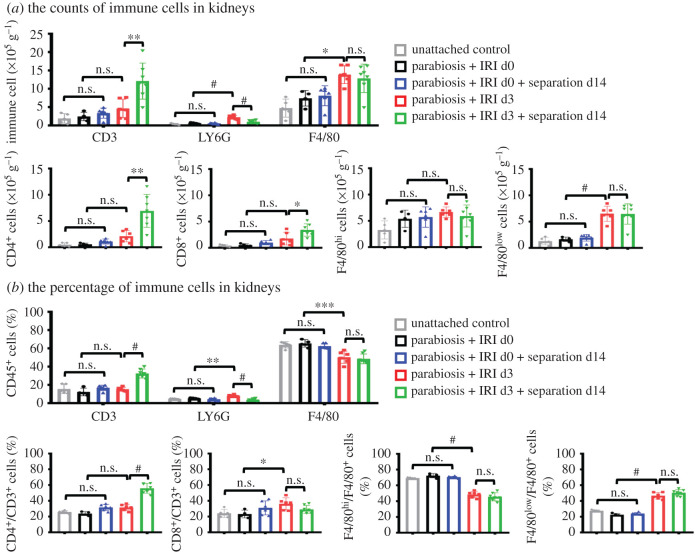
Parabiosis and separation surgery do not alter the proportion of immune cells in kidneys. (*a*) The analyses by flow cytometry of the number of T lymphocytes (total, CD4^+^, CD8^+^ T lymphocytes), neutrophils and macrophages (total, F4/80^hi^, F4/80^low^ macrophages) from WT kidneys in each group. (*b*) The analyses by flow cytometry of the percentage of CD3^+^ T lymphocytes, LY6G^+^ neutrophils and F4/80^+^ macrophages among CD45^+^ cells from WT kidneys (above). The analyses by flow cytometry of the percentage of CD4^+^ cells among CD3^+^ T lymphocytes, CD8^+^ cells among CD3^+^ T lymphocytes, F4/80^hi^ cells among F4/80^+^ macrophages and F4/80^low^ cells among F4/80^+^ macrophages from WT kidneys (below). *n* = 4–7 per group. Values were means ± s.d. n.s., no significance; *p < 0.05; **p < 0.01; ***p < 0.001; ^#^*p* < 0.0001.

After the construction of parabiosis, neovascularization appears resulting in the sharing of blood circulation between the two mice [23]. Flow cytometry analyses showed that at day 28 after parabiosis approximately 50% of the blood leucocytes in WT mice were chimeric cells from GFP mice. At day 14 after parabionts dissociation, nearly half of the GFP^+^ leucocytes in the blood of recipients disappeared in both injured and uninjured separants, indicating kidney damage did not alter the chimerism rate of blood cells (figure 2*a*). Partner-derived cells were also detected in kidneys of WT mice. When the kidneys were damaged, the percentage of chimerism of leucocytes in the injured kidneys increased obviously compared with that in uninjured controls, no matter parabionts or separants (figure 2*b*). PAS staining showed that renal tubular dilation and tubular atrophy occurred at day 3 and day 17 after IRI, respectively (figure 2*c*). The protein expression of kidney injury molecule 1 (Kim-1) in IRI mice was higher than that in non-IRI mice (figure 2*d*). Masson staining confirmed that collagen deposition was greater in renal interstitium after IRI compared with the uninjured control group (figure 2*e*). The same result was observed by immunofluorescence-labelled α-SMA (a specific marker of myofibroblasts) (figure 2*f*). Comparing the four surgery groups with each other, we found that the number and percentage of T lymphocytes kept at high levels at CKD phase (day 17 after IRI), while neutrophils kept at high levels at AKI phase (day 3 after IRI) (figure 3*a*). Bonavia *et al*. reported that T lymphocytes mainly occur in the chronic stage of kidney injury, and neutrophils generally appear in the acute stage of kidney injury [8,26], which was consistent with our data. The flow cytometry analyses also showed that the number of macrophages in kidneys of the injured mice at day 3 and 17 after IRI was significantly higher than that of the uninjured mice (figure 3*a*). The same results were observed by the immunofluorescence-labelled CD3, F4/80 and LY6G (electronic supplementary material, figure S1). The above data proved that the parabiosis, separation and IRI models were successfully established.

### 3.3. Parabiosis and separation surgery do not alter the proportion of immune cells in kidneys

In order to verify whether parabiosis and separation surgery could cause extra immune response, we measured the proportion of immune cells in kidneys among CD45^+^ cells by flow cytometry, comparing parabionts and separants to unattached control mice with the same genetic background. The gating strategies are shown in the electronic supplementary material, figure S2. We found that the proportion of T lymphocytes (CD45^+^CD3^+^), neutrophils (CD45^+^CD11b^+^LY6G^+^) and macrophages (CD45^+^CD11b^+^F4/80^+^) in kidneys of unattached WT mice were 18%, 4% and 65%, respectively, which was consistent with previous studies [27–30]. Flow cytometry analyses revealed that the number and percentage of leucocytes in kidneys of parabionts and separants without IRI injury have no significance compared to unconnected control mice (figure 3*a*,*b*), indicating that the composition of immune cells residing in the normal kidneys was not affected by the operation of parabiosis and separation.

### 3.4. Peripheral T lymphocytes can survive in normal and damaged kidneys for a long time and are continuously recruited to kidneys during the AKI-CKD progression

**Figure 4.**
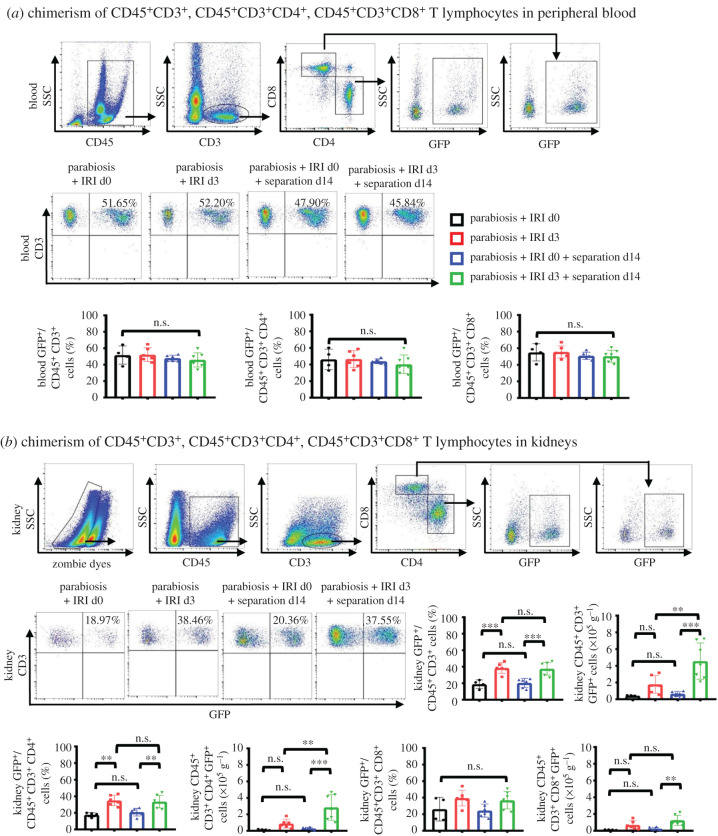
Peripheral T lymphocytes can survive in kidneys for a long time. (*a*) The analyses by flow cytometry of the percentage of GFP^+^ cells among CD3^+^, CD3^+^CD4^+^ and CD3^+^CD8^+^ T lymphocytes in peripheral blood from different groups. Gating strategies were showed. (*b*) The analyses by flow cytometry of the percentage of GFP^+^ cells among CD3^+^, CD3^+^CD4^+^, CD3^+^CD8^+^ T lymphocytes and the number of CD45^+^CD3^+^GFP^+^, CD45^+^CD3^+^CD4^+^GFP^+^, CD45^+^CD3^+^CD8^+^GFP^+^ cells in kidneys from each group. Gating strategies were showed. *n* = 4–7 per group. Values were means ± s.d. n.s., no significance; **p < 0.01; ***p < 0.001.

After parabiosis at day 28, T lymphocytes demonstrated an average per cent chimerism equal to 51.65% in the blood of uninjured parabionts. After separation or IRI, the chimerism rate did not change significantly. Similar trends were observed for CD4^+^ T lymphocytes and CD8^+^ T lymphocytes. The above results suggest that the T lymphocytes from the partners, including two sub-types, can survive for a long time in the blood (figure 4*a*). In kidneys of uninjured parabionts and separants, there was also no significant difference in the chimeric rate of T lymphocytes. The same results were observed in injured parabionts and separants, which suggests that peripheral-derived T lymphocytes can survive for more than 14 days in normal and damaged kidneys. The percentage and number of GFP^+^ T lymphocytes in kidneys of injured mice were significantly higher than those of the undamaged mice, indicating that more peripheral T lymphocytes were recruited and survived into kidneys after IRI. In addition, the absolute number of peripheral GFP^+^ T cells in kidneys of injured separants was significantly higher than that of injured parabionts, which revealed that T cells could continuously migrate to or be survived in kidneys in the process of AKI-CKD. The dynamic changes of peripheral CD4^+^ and CD8^+^ T lymphocytes were similar to those of peripheral CD3^+^ T lymphocytes, but the number of CD4^+^GFP^+^ T lymphocytes (2.41 × 10^5^ ± 0.52 × 10^5^ cells/g tissue) was greater in kidneys at day 17 after IRI than that of CD8^+^GFP^+^ T lymphocytes (1.25 × 10^5^ ± 0.23 × 10^5^ cells/g tissue) (figure 4*b*). Compared with CD8^+^ T cell, CD4^+^ T cell is a more important mediator of ischaemic acute renal failure [31].

### 3.5. Peripheral neutrophils exhibit a short lifespan in normal and damaged kidneys

**Figure 5.**
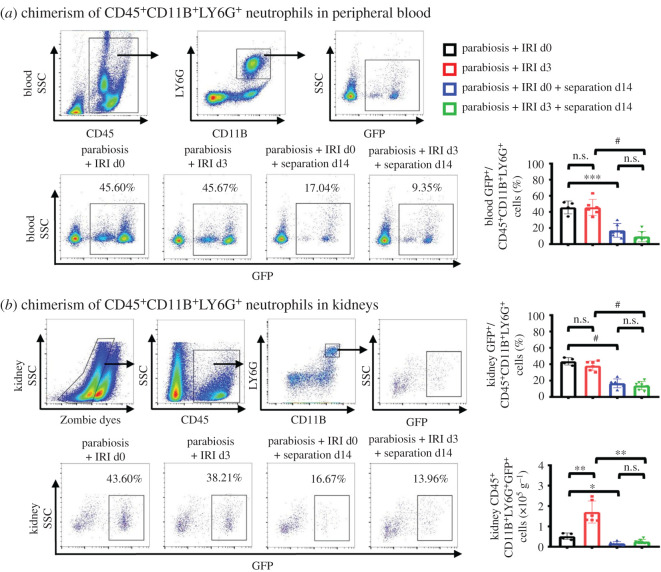
Peripheral neutrophils have a short survival time in kidneys. (*a*) The percentage of GFP^+^ cells among CD11B^+^LY6G^+^ neutrophils analyses by flow cytometry in peripheral blood from different groups of mice. Gating strategies were showed. (*b*) The analyses by flow cytometry of the percentage of GFP^+^ cells among CD11B^+^LY6G^+^ neutrophils and the number of CD45^+^ CD11B^+^LY6G^+^GFP^+^ cells in kidneys. Gating strategies were showed. *n* = 4–7 per group. Values were means ± s.d. n.s., no significance; **p < 0.01; ***p < 0.001; ^#^*p* < 0.0001.

We found no matter the parabionts underwent IRI surgery or not, there was no significant difference in the chimerism of CD45^+^CD11B^+^LY6G^+^ neutrophils in the blood. However, at day 14 after the separation of the conjoined pairs, the chimerism of neutrophils in blood was significantly reduced, indicating that neutrophils are a type of short-lived cells in the peripheral circulation system (figure 5*a*). In kidneys, we found that the infiltrating neutrophils were mostly derived from peripheral circulation regardless of whether the kidneys were damaged. After eliminating the source of GFP^+^ cells from the partners for 14 days, most of the GFP^+^ cells in kidneys disappeared (figure 5*b*), suggesting that the neutrophils were recruited into kidneys only at acute phase after renal injury and survived for a short time in kidneys.

### 3.6. The survival time of peripheral-derived macrophages in injured kidneys was longer than that in quiescent kidneys

**Figure 6.**
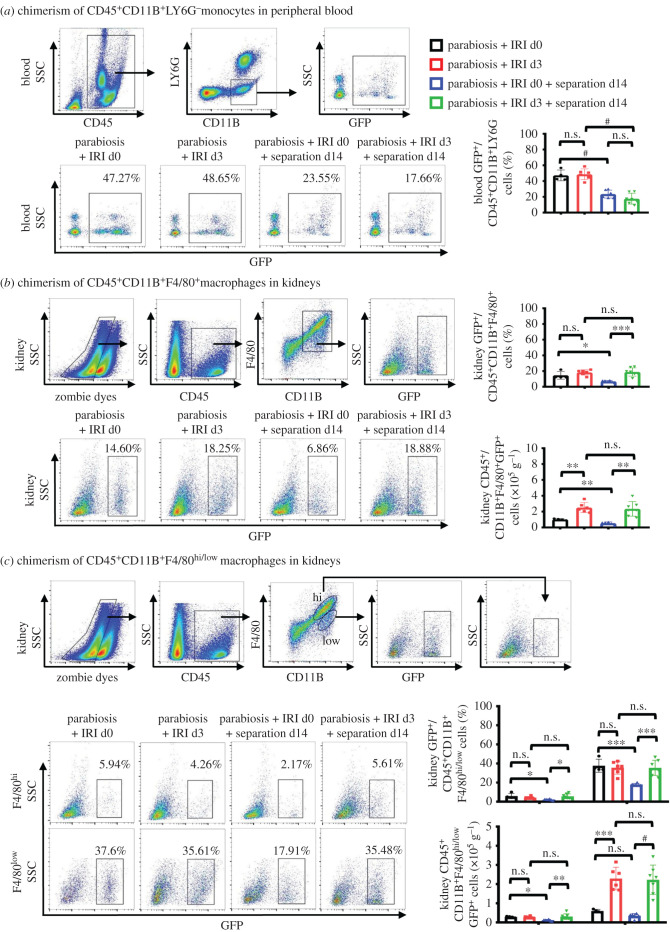
The renal inflammatory micro-environment contributes to the lifespan prolongation of macrophages from peripheral circulation in kidneys, and peripheral monocytes can be transformed into tissue-resident macrophages. (*a*) The analyses by flow cytometry of the percentage of GFP^+^ cells among CD11B^+^LY6G^−^ monocytes in peripheral blood from different groups of mice. Gating strategies were showed. (*b*) The analyses by flow cytometry of the percentage of GFP^+^ cells among CD45^+^CD11B^+^F4/80^+^ macrophages and the number of CD45^+^CD11B^+^F4/80^+^GFP^+^ cells in kidneys from each groups of mice. (*c*) The analyses by flow cytometry of the percentage of GFP^+^ cells among CD45^+^CD11B^+^F4/80^hi\low^ macrophages and the number of CD45^+^CD11B^+^ F4/80^hi\low^ GFP^+^ cells in kidneys from each groups of mice. Gating strategies were showed. *n* = 4–7 per group. Values were means ± s.d. n.s., no significance; *p < 0.05; **p < 0.01; ***p < 0.001; ^#^*p* < 0.0001.

At day 28 after conjoined symbiosis, nearly half of the myeloid monocytes (CD45^+^CD11b^+^LY6G^−^) in the blood of matched WT mice showed GFP positive, confirming that the monocytes exchange completely in the matched two mice. However, after the conjoined pairs were dissociated, the chimeric rate of monocytes in the blood decreased by about half at day 14 after dissociation (figure 6*a*). Further, the total number and percentage of GFP^+^ macrophages in the uninjured parabionts' kidneys were reduced at day 14 after dissociation, indicating that a portion of the peripheral-derived macrophages disappeared under normal homeostasis conditions in 14 days. Interestingly, the number and percentage of GFP^+^ macrophages in kidneys of injured separants showed no significant difference compared with injured parabionts, but increased compared to uninjured separants, which suggests that the renal inflammatory micro-environment contributes to recruiting more peripheral macrophages into kidneys and prolonging the lifespan of peripheral-derived macrophages in kidneys (figure 6*b*). Similar results were observed in F4/80^hi^ and F4/80^low^ macrophages, as shown in figure 6*c*. In addition, in kidneys of uninjured or injured parabionts, F4/80^low^GFP^+^ macrophages accounted for about 40% (among F4/80^low^ macrophages), while only a small part of F4/80^high^ macrophages presented GFP^+^. Based on these results, we speculated that whether the kidneys were normal or damaged, the majority of F4/80^low^ macrophages were differentiated from peripheral monocytes, while a small proportion of F4/80^hi^ macrophages were differentiated from peripheral monocytes. Most of F4/80^hi^ macrophages might depend on their own proliferation.

### 3.7. Excretion of related survival factors and proliferation of peripheral -derived cells may contribute to prolonging the survival time of inflammatory cells in kidneys

**Figure 7.**
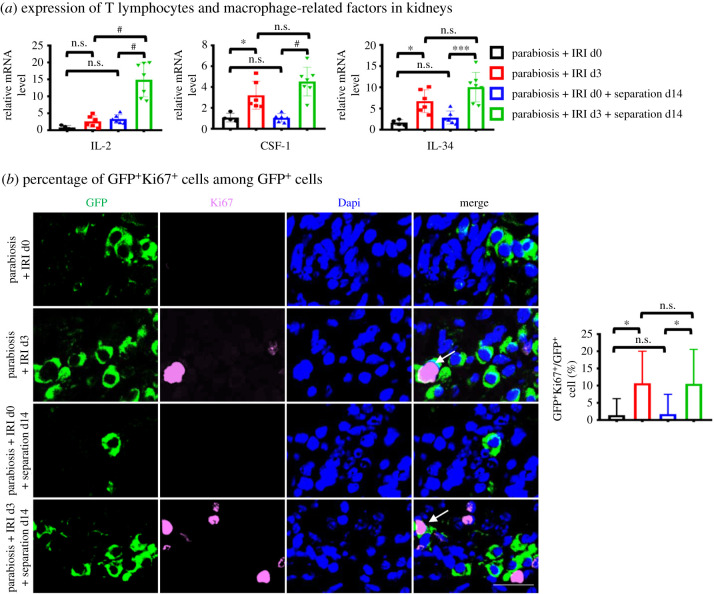
The expression levels of survival factors of T cells and macrophages increase and a small number of peripheral cells can proliferate in kidneys after IRI. (*a*) Q-PCR for levels of T lymphocytes growth factors IL-2 and macrophage growth factors CSF-1, IL34. (*b*) Representative images and double-positive analyses for immunofluorescence-labelled GFP (green) and Ki67 (red). Scale bar = 20 µm. *n* = 4–7 per group. Values were means ± s.d. n.s., no significance; *p < 0.05; ***p < 0.001; ^#^*p* < 0.0001.

Interleukin-2 (IL-2) is essential for the proliferation of T cells, production of effector cell and memory cell [32]. We found the mRNA expression level of IL-2 in kidneys of CKD stage was higher than that of AKI stage or quiescent stage (figure 7*a*). IL-34 and CSF-1 share the same receptor, and both mediate the survival, proliferation and function of macrophages [33,34]. The mRNA expression levels of CSF-1, and IL-34 were increased after IRI (figure 7*a*), which suggests a formation of a pro-proliferative and pro-survival micro-environment for macrophages in injured kidneys. Ki67 is a well-known proliferation marker used to assess cell proliferation. We found co-staining of GFP and Ki67 in injured kidneys showed that peripheral-derived cells could self-proliferate in injured kidneys (figure 7*b*).

## 4. Discussion

Inflammation and leucocyte recruitment are key mediators in all stages of kidney injury. Soon after the injury of endothelial or tubular epithelial cells, kidney resident inflammatory cells are activated, followed by recruitment and infiltration of different subsets of leucocytes. Almost all immune cells are involved in the pathogenesis of AKI [8,15]. Thus, the dynamics of immune cells in blood and kidney have aroused great interest.

However, the survival time of peripheral-derived immune cells in the kidney, especially in the injured kidney, is rarely reported. To explore this issue, an appropriate model needs to be implemented urgently. In this study, we combined parabiosis, IRI surgery and separation. Parabiosis is a surgical combination of two organisms that allows for shared blood circulation [35–37]. It has been employed to investigate the dynamics of macrophages in the central nervous system [38], the mechanisms of stem cell ageing [39], as well as the effect of circulating factors on glomerular damage [40]. Parabiotic partners share their circulating antigens, so there is no adverse immune response [23,41]. In this model, one WT mouse was surgically linked to another GFP mouse to achieve parabiosis. After about four weeks, flow cytometry analyses of blood in WT mice revealed the presence of chimeras, indicated by about half of GFP positive cells. Various experimental models have been created to imitate the complexity and diversity of AKI [42]. One of the main causes of AKI is renal IRI [43–45]. Therefore, the left renal pedicle of each WT mouse was clamped for 30 min to achieve IRI-induced AKI in our study. We could trace the immune cells migrating to damaged kidneys from the blood of GFP mice by flow cytometry as the cells presented GFP positive. The chimeric cells of the symbiotic partners would continuously enter the peripheral blood of WT mice and continue to exist, making it impossible to observe the life cycle of these cells. Thus, parabionts were separated to eliminate the source of renewal of GFP^+^ cells. At day 14 after dissociation, the percentage and number of GFP^+^ cells among different immune cells that survived in the injured kidneys of WT mice were measured by flow cytometry to roughly judge their lifespan.

In this study, we revealed that different immune cells exhibited different life cycles in the kidney. In a normal physiological status, peripheral lymphocytes could survive longer, whereas peripheral neutrophils and macrophages survived shorter in the kidney. Most GFP positive neutrophils and nearly half of GFP positive macrophages perished at 14 days after dissociation, while GFP positive lymphocytes did not change obviously. These findings were consistent with the conclusions of previous studies. T lymphocytes in the thymus are developed from common lymphoid progenitor cells, and they can live as long as months or even years in different tissues. After contact with antigen, T lymphocytes can differentiate into memory T cells with different migration patterns and life cycles [20,21,46]. Of course, it is possible that chimeric T cells from the spleen or bone marrow account for the continued presence following injury. Assays of GFP positive cells in spleen and bone marrow following separation would be very helpful to evaluate this possibility in future studies. Neutrophils are short-lived in immune cells and are recognized as originating from neutrophil progenitor cells in the bone marrow. In general, the average lifespan of neutrophils varies from hours to days [47–49]. Bone marrow-derived monocytes are circulating precursors that supplement macrophage populations in surrounding tissues during homeostasis [50]. Studies have shown that the residence time of monocytes in the blood is 2 to 4 days [51,52], and macrophages derived from monocytes in the eye tissue have a lifespan of several weeks [50].

Surprisingly, we found that the number and proportion of peripheral-derived macrophages after kidney injury remained unchanged at 14 day after dissociation, which was inconsistent with the results observed in normal kidneys. We speculate that the reason is that renal micro-environment promotes the survival of peripheral-derived macrophages via self-proliferation after settling in the kidney. Macrophages are a highly heterogeneous population, and two subgroups of macrophages have been identified in some studies: (i) F4/80^high^ expression population is considered to be tissue-resident macrophages derived from the yolk sac and fetal liver in embryonic life and (ii) F4/80^low^ expression population is defined as inflammatory infiltrating macrophages derived from the monocyte pool in the peripheral circulation [27,53]. However, conclusions regarding the enduring sources of renewal for F4/80^high^ macrophages in the kidney are still controversial. Two potential sources of F4/80^high^ macrophage are blood mononuclear phagocyte cells and *in situ* renewal [53,54]. Our results indicated that F4/80^high^ macrophages were mainly originated from *in situ* proliferation, and a small part were differentiated from circulating monocytes. Different from peripheral-derived macrophages, the inflammatory state of the kidney did not manifestly affect the life cycle of peripheral neutrophils, suggesting that neutrophils are short-lived and unable to self-renew in the kidney. The life cycle of peripheral lymphocytes is relatively long (longer than 14 days), so the data at day 14 after dissociation cannot fully demonstrate whether the inflammatory state of the kidney affects the life cycle of lymphocytes.

In previous studies, myeloid cells have been generally regarded as short-lived cells. Peripheral myeloid neutrophils mostly survive for 19 h to 4 days [47,48] and myeloid monocytes survive for less than 7 days [51,52]. Therefore, it is predicted that at 14 days after parabiosis and dissociation, the chimerism rate of myeloid monocytes and myeloid neutrophils in peripheral blood should drop close to 0%. However, our experiment results showed that although both neutrophils and monocytes decreased at 14 days after dissociation compared with that prior to the dissociation, some of them still existed in peripheral blood. Wright *et al*. confirmed that at 7, 10 and 22 weeks after the parabiotic mice were dissociated, the chimerism rate of neutrophils in the peripheral blood was not close to 0%. It might be related to that haematopoietic stem cells (HSCs) were implanted into the bone marrow of the partner by parabiosis [55]. According to another literature, at 6–24 weeks after separation, the myeloid cells from another mouse still survive in the peripheral blood. These cells may be long-lived partner-derived lymphoid progenitors, expressing both myeloid and lymphoid lineage markers [56]. Therefore, we speculate that at day 14 after dissociation in this experiment, these survived peripheral myeloid cells might be differentiated from HSCs of GFP mice implanting into the bone marrow of WT mice or be one special type of lymphoid cells. Further studies will be required for more evidence.

In conclusion, our work demonstrated that T lymphocytes, neutrophils and peripheral-derived macrophages exhibit different lifespans in the kidney. In both normal and damaged kidneys, peripheral T lymphocytes are long-lived, and peripheral neutrophils are short-lived. Inflammatory micro-environment leads to prolonging the survival time of peripheral-derived macrophages in the kidney. Extended dissociation time should be applied in our future research, so as to track immune cells for a longer term and clarify their survival characteristics.

## Data accessibility

All data are included in the manuscript figures and electronic supplementary material.
